# RNF8 ubiquitinates RecQL4 and promotes its dissociation from DNA double strand breaks

**DOI:** 10.1038/s41389-021-00315-0

**Published:** 2021-03-05

**Authors:** Qunsong Tan, Kaifeng Niu, Yuqi Zhu, Zixiang Chen, Yueyang Li, Mengge Li, Di Wei, Adayabalam S. Balajee, Hongbo Fang, Yongliang Zhao

**Affiliations:** 1 Key Laboratory of Genomic and Precision Medicine, Beijing Institute of Genomics, Chinese Academy of Sciences, Beijing, 100101 China; 2 China National Center for Bioinformation, Beijing, 100101 China; 3 University of Chinese Academy of Sciences, Beijing, 100049 China; 4 REAC/TS, Oak Ridge Associated Universities, Oak Ridge Institute for Science and Education, 1299 Bethel Valley Road, Oak Ridge, TN 37830 USA

**Keywords:** Double-strand DNA breaks, Homologous recombination

## Abstract

Ubiquitination-dependent DNA damage response (DDR) signals play a critical role in the cellular choice of DNA damage repair pathways. Human DNA helicase RecQL4 participates in DNA replication and repair, and loss of RecQL4 is associated with autosomal recessive genetic disorders characterized by genomic instability features. In an earlier study, RecQL4 was isolated as a stable complex that contained two ubiquitin ligases of the N-end rule (UBR1 and UBR2). However, it is unknown whether or not RecQL4 ubiquitination status is critical for its DNA repair function. Here, we report that RecQL4 directly interacts with RNF8 (a RING finger ubiquitin E3 ligase), and both co-localize at DNA double-strand break (DSB) sites. Our findings indicate that RNF8 ubiquitinates RecQL4 protein mainly at the lysine sites of 876, 1048, and 1101, thereby facilitating the dissociation of RecQL4 from DSB sites. RecQL4 mutant at ubiquitination sites had a significantly prolonged retention at DSBs, which hinders the recruitment of its direct downstream DSB repair proteins (CtIP & Ku80). Interestingly, reduced DSB repair capacity observed in RecQL4 depleted cells was restored only by the reconstitution of wild-type RecQL4, but not the ubiquitination mutant. Additionally, RecQL4 directly interacts with WRAP53β that is known to recruit RNF8 to DSBs and WRAP53β enhances the association of RecQL4 with RNF8. WRAP53β silencing resulted in a nearly diminished recruitment of RNF8 to DSBs and in a greatly attenuated dissociation of RecQL4 from the DSB sites. Collectively, our study demonstrates that the ubiquitination event mediated by RNF8 constitutes an essential component for RecQL4’s function in DSB repair.

## Introduction

Posttranslational modifications of proteins, including non-degrading ubiquitination, precisely control the assembly or disassembly of DNA damage repair (DDR) proteins at DNA damage sites^1,2^. Ubiquitination, a covalent attachment of highly conserved single ubiquitin or complex polyubiquitin chains to lysine sites of proteins, provides quality controls through mediating DNA damage repair activities, and has been demonstrated to be a critical modification for the cellular choice of DNA repair pathways^3,4^. RNF8 and RNF168 are two most extensively studied E3 ligases, and their dependent DDR signals have been shown to suppress homologous recombination (HR) while promoting non-homologous end joining (NHEJ) pathway by recruiting 53BP1 and RAP80^3,5^. Upon generation of DNA double-strand breaks (DSBs), ataxia telangiectasia-mutated (ATM) kinase phosphorylates MDC1 and subsequently promotes the recruitment of WRAP53β (WD40 encoding antisense to p53) which targets RNF8 to DNA damage sites^6,7^. Additionally, RNF168 coordinates with RNF8 and amplifies the ubiquitin signal required for retention of two decisive factors (53BP1 and BRCA1) in the choice between NHEJ and HR repair pathways^8^. Meanwhile, NHEJ pathway protein Ku80 is also ubiquitinated by these two E3 ligases, and removed from chromatin in a valosin-containing protein (VCP)/p97-dependent manner^9^. All these findings suggest a critical role of ubiquitination modification in modulating the activity of DNA damage repair proteins.

RecQL4 is one of the five human DNA helicases and its mutational inactivation results in autosomal recessive genetic disorders^10,11^. In relative to other members of RecQ helicases, RecQL4 was shown to be recruited to DSB sites at much earlier time-point relative to other RecQ helicases (BLM, WRN), and participate in DSB repair through association with multiple downstream proteins, including MRN complex, CtIP, and Ku70/Ku80, etc^12–14^. One recent report demonstrated that CDK1/2-mediated phosphorylation of RecQL4 not only regulates the choice of DNA repair pathway in a cell cycle-dependent manner but also promotes RecQL4 ubiquitination by DDB1-CUL4A E3 ubiquitin ligase leading to an enhanced activity of RecQL4 in DSB repair^15^. It remains to be investigated as to whether or not other ubiquitin E3 ligases ubiquitinate RecQL4 protein and how this modification regulates its DNA repair function. Here, we provide evidence for the first time that RNF8 ubiquitinates RecQL4 protein and promotes its dissociation from DSB sites. Additionally, WRAP53β directly associates with RecQL4, and enhances the interaction between RNF8 and RecQL4. Depletion of WRAP53β led to a defective recruitment of RNF8 to DSBs and further a prolonged retention of RecQL4 protein at DSBs. Overall, our findings illustrate a critical role of RNF8-mediated RecQL4 ubiquitination in DSB repair process.

## Results

### RecQL4 physically interacts with RNF8

**Fig. 1:**
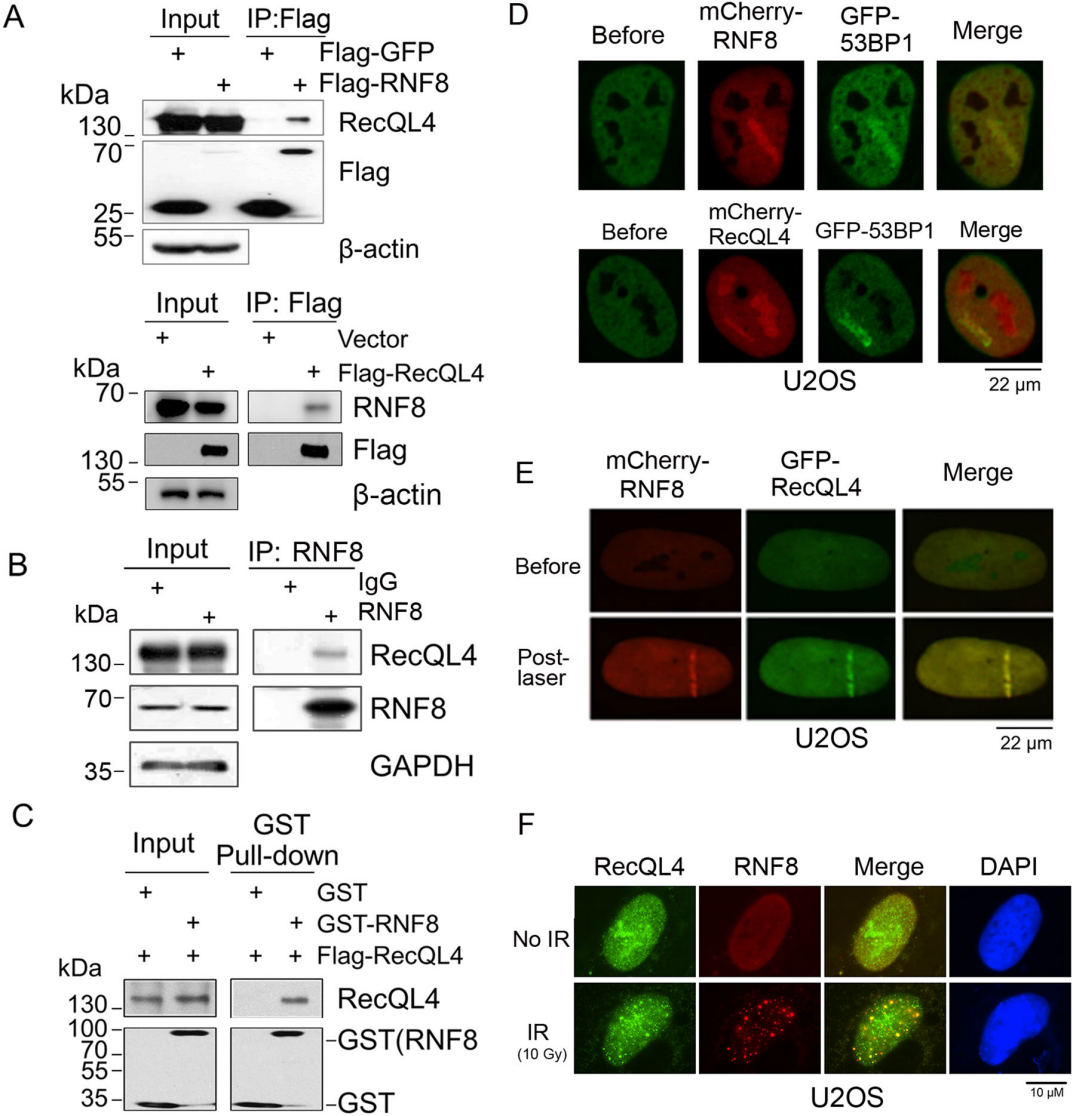
RecQL4 physically interacts with RNF8. **A** Endogenous RecQL4 or RNF8 were detected in anti-Flag immunoprecipitated fractions from Flag-RNF8/Flag-RecQL4 transfected U2OS cells by western blotting analysis. Flag-GFP/Empty vector-transfected cells were used as negative controls. **B** Interaction between endogenous RecQL4 and RNF8. Immunoprecipitated fraction from U2OS lysate was prepared using anti-RNF8 antibody (14112-1-AP, Proteintech), followed by western blotting analysis with anti-RecQL4 (25470002, SDIX) or RNF8 antibodies (sc-271462, Santa cruz). RecQL4 protein was detected in the RNF8 immunoprecipitated complex. **C** Direct interaction between RecQL4 and RNF8 was demonstrated by an in vitro pull-down assay. Purified recombinant GST-RNF8 was immobilized on Glutathione resin and incubated with purified Flag-RecQL4 in the IP buffer, and the bound proteins were examined by western blotting. **D** Colocalization of GFP-tagged 53BP1 and mCherry-tagged RNF8/RecQL4 at DSB track induced by UV micro-point laser. **E** Co-localization of mCherry-RNF8 and GFP-RecQL4 at DSB track induced by UV micro-point laser. **F** Co-localization of endogenous RNF8 and RecQL4 after X-ray irradiation. U2OS cells were exposed to 10 Gy of X-ray irradiation (25 mA, 160 kV; dose rate 0.995 Gy/min, X-RAD RS2000, Rad Source, USA), and fixed with 4% paraformaldehyde at 3.5 h post treatment. Indirect immunostaining was performed using primary anti-RecQL4 and RNF8 and fluorescence-dye conjugated secondary antibodies. After counterstaining with DAPI, images were captured using a fluorescence microscope (Leica DM5000 Microsystems).

DDB1–CUL4A E3 complex has been shown to ubiquitinate RecQL4 and promote its accumulation at DNA damage sites^15^. To search for other potential ubiquitin E3 ligase(s) that can modulate RecQL4 activity, we have screened a number of E3 ligases that have been reported to participate in the repair of DNA damage^16^, and identified RNF8 as an interaction partner of RecQL4. To verify and validate the interaction, U2OS cells were transfected with either Flag-RecQL4 or Flag-RNF8. The Flag pull-down protein complex was then tested by western blotting using RecQL4 and RNF8 specific antibodies. The endogenous level of RecQL4 or RNF8 was detected in the Flag-RNF8 and Flag-RecQL4 pull-down complex, respectively (Fig. 1A). Consistently, endogenous RecQL4 could be immunoprecipitated by anti-RNF8 antibody (Fig. 1B), confirming an interaction between RNF8 and RecQL4.

The nature of their interaction was then tested by an in vitro pull-down assay. Recombinant proteins of GST-RNF8 and Flag-RecQL4 were expressed and purified from IPTG-induced *E.coli* BL21. Purified GST-RNF8 protein was immobilized on glutathione Sepharose beads and then incubated with recombinant Flag-RecQL4 protein. Consistent with the co-IP results, RecQL4 was pulled down with GST-RNF8 (Fig. 1C), but not by GST alone, suggesting a direct interaction between RecQL4 and RNF8.

To precisely identify the interacting domain (s) of RecQL4 with RNF8, we used the Flag-tagged truncated recombinant RecQL4, including N-terminal (NT, 1–475 aa), helicase domain (HD, 476–824 aa), and C-terminal domains (CT, 825–1206 aa) for the co-immunoprecipitation (co-IP) assays followed by western blotting with anti-RNF8 antibody. The results indicated that RNF8 can be efficiently pulled down by all three different RecQL4 domains (Supplementary Fig. 1). As RecQL4 interacts with RNF8 in vivo and in vitro, we then tested their real-time recruitment at DSB sites induced by micro-point laser. Using 53BP1 as a positive DSB maker, RNF8 or RecQL4 was shown to be recruited to DSB track induced by micro-point laser (Fig. 1D). Further results from both treatments of micro-point laser and X-ray irradiation demonstrated that RNF8 co-localizes with RecQL4 at DSBs (Fig. 1 E, F), supporting their physical interaction.

### RNF8 ubiquitinates RecQL4 in vivo and in vitro

**Fig. 2:**
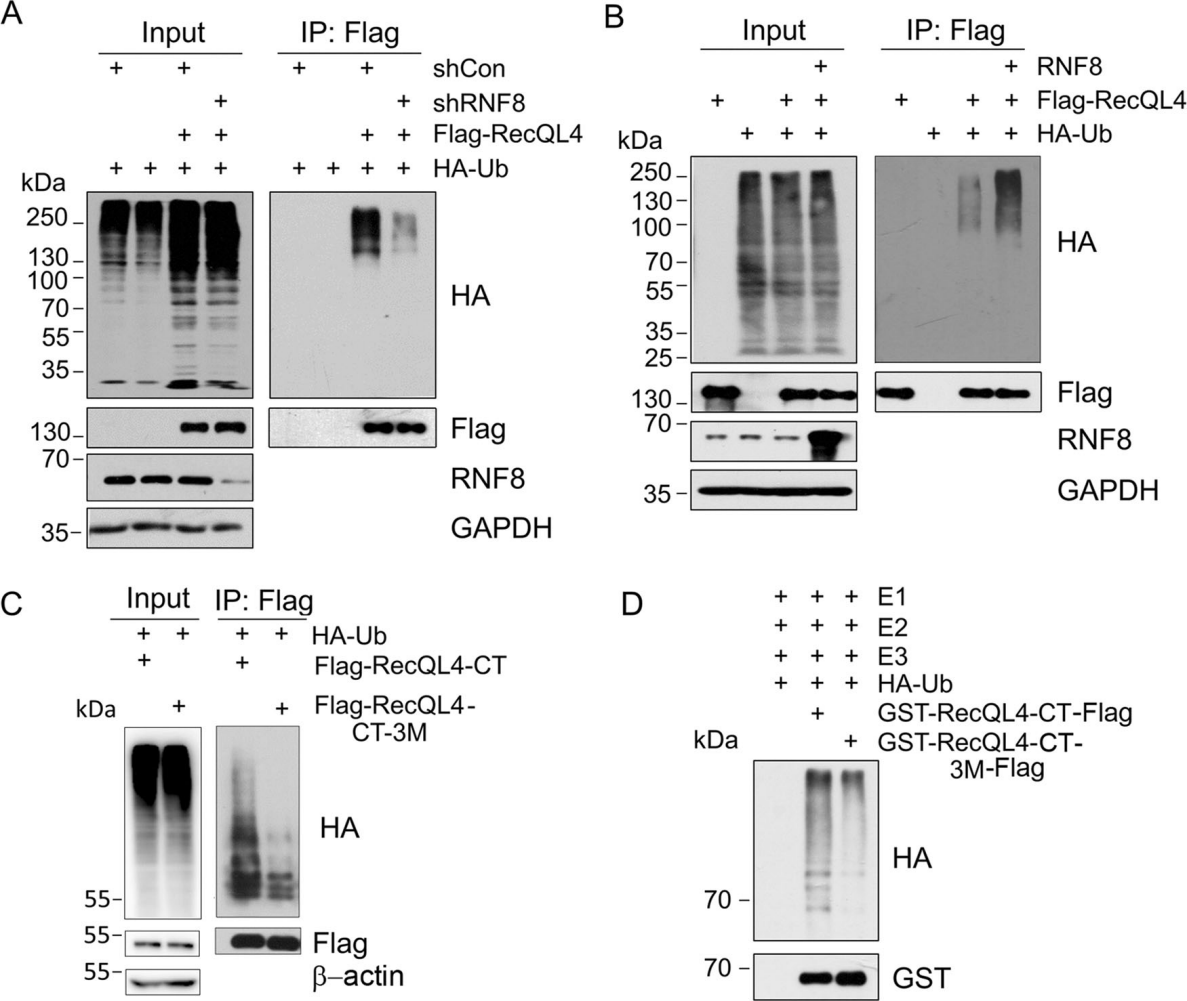
RecQL4 is the substrate of RNF8. **A** RecQL4 ubiquitination level was decreased upon RNF8 depletion. U2OS cells were transfected with Flag-RecQL4 plasmid, followed by control/RNF8 shRNA and HA-Ub plasmids. Immunoprecipitation was performed on the cell lysates using anti-Flag (M2) beads, and RecQL4 ubiquitination level was determined by western blotting analysis with anti-HA antibody. **B** RNF8 promotes RecQL4 ubiquitination in vivo. U2OS cells were transfected with Flag-RecQL4, HA-Ub, and RNF8 plasmids. Empty vector was used as control. **C**, **D** RecQL4 CT mutant (3M: K876R-K2048R-K1101R) showed a markedly decreased ubiquitination level relative to wild-type RecQL4 CT in both in vivo and in vitro ubiquitination assays.

The observation of physical interaction between RecQL4 and RNF8 led us to examine whether or not RNF8 catalyzes the ubiquitination of RecQL4 protein. For this, RNF8 expression was first silenced in U2OS cells by adenoviral-mediated shRNA for RNF8 followed by ectopic expression of Flag-RecQL4 and HA-Ub in both scramble shRNA control (shCon) or RNF8-knockdown (shRNF8) cells. After Flag-RecQL4 pull-down with Flag M2 beads, ubiquitination level of RecQL4 was examined by western blotting with anti-HA antibody against HA-Ub. The result showed that RecQL4 ubiquitination was markedly decreased in RNF8-depleted cells (Fig. 2A). Conversely, ectopic expression of RNF8 substantially enhanced the ubiquitination of RecQL4 in U2OS cells (Fig. 2B). These cross-validating experiments convincingly demonstrated the ubiquitination of RecQL4 by RNF8.

The targeted domains of RecQL4 by RNF8 were then examined using plasmids expressing different domains of Flag-tagged RecQL4 protein (NT, HD, and CT) by co-transfection of each individual vector with HA-Ub in U2OS cells. Only helicase and C-terminal domains showed ubiquitination in vivo (Supplementary Fig. 2A). We then screened the ubiquitinated lysine sites potentially targeted by RNF8. Each of HD or CT expressing plasmids contained one mutated lysine site (K to R). The results showed that the C terminus with K876R, K1048R, or K1101R mutations resulted in a substantially decreased ubiquitination level relative to wild-type C-terminal protein (Supplementary Fig. 2B, C), suggesting that these three C-terminal lysine residues are the main potential target sites by RNF8. In support, C-terminal RecQL4 protein with triple mutations at these sites (3M, K876R, K1048R, and K1101R) exhibited a significantly decreased ubiquitination level (Fig. 2C).

As RNF8 primarily targets C terminus of RecQL4, we then chose C-terminal RecQL4 protein (WT & 3M) for an in vitro ubiquitination assay to validate the in vivo findings following the previously reported procedure^17^. Purified recombinant RNF8 and RecQL4-CT domain or its mutant (3M) were incubated in the reaction buffer in the presence of Ub, purified E1 (UBA1), and E2 (UbcH5c) enzymes. The results illustrated that RNF8 efficiently catalyzes RecQL4 ubiquitination, but the level was substantially decreased in the RecQL4-CT mutant (3M) relative to the wild type RecQL4-CT protein (Fig. 2D).

Observation of RNF8-dependent ubiquitination of RecQL4 prompted us to identify the types of ubiquitin chain linkage on RecQL4 protein. Previous findings have demonstrated that ubiquitin chains linked at K48 and K63 are observed in the vicinity of DNA breaks^18^, and RNF8-mediated K63 chains are particularly important in the recruitment of downstream DDR repair proteins, such as RAP80 and 53BP1^19^. In this study, a series of HA-tagged Ub mutants containing only one lysine site with all other lysine residues mutated to arginine were generated and individually co-transfected with Flag-RecQL4 in control and RNF8-knockdown U2OS cells for 24 h. The cell lysates were immunoprecipitated with anti-Flag M2 beads. Each K-linked ubiquitination level of RecQL4 was then analyzed by western blotting with anti-HA antibody. The results showed that RNF8 knockdown substantially reduced the level of K6, K27, and K29-linked ubiquitin conjugate on RecQL4 (Supplementary Fig. 3), suggesting that ubiquitin chain formation at the Lys6 and 27 sites are the major types of RecQL4 polyubiquitination mediated by RNF8.

### RNF8-mediated RecQL4 ubiquitination is required for its dissociation from DSBs

**Fig. 3:**
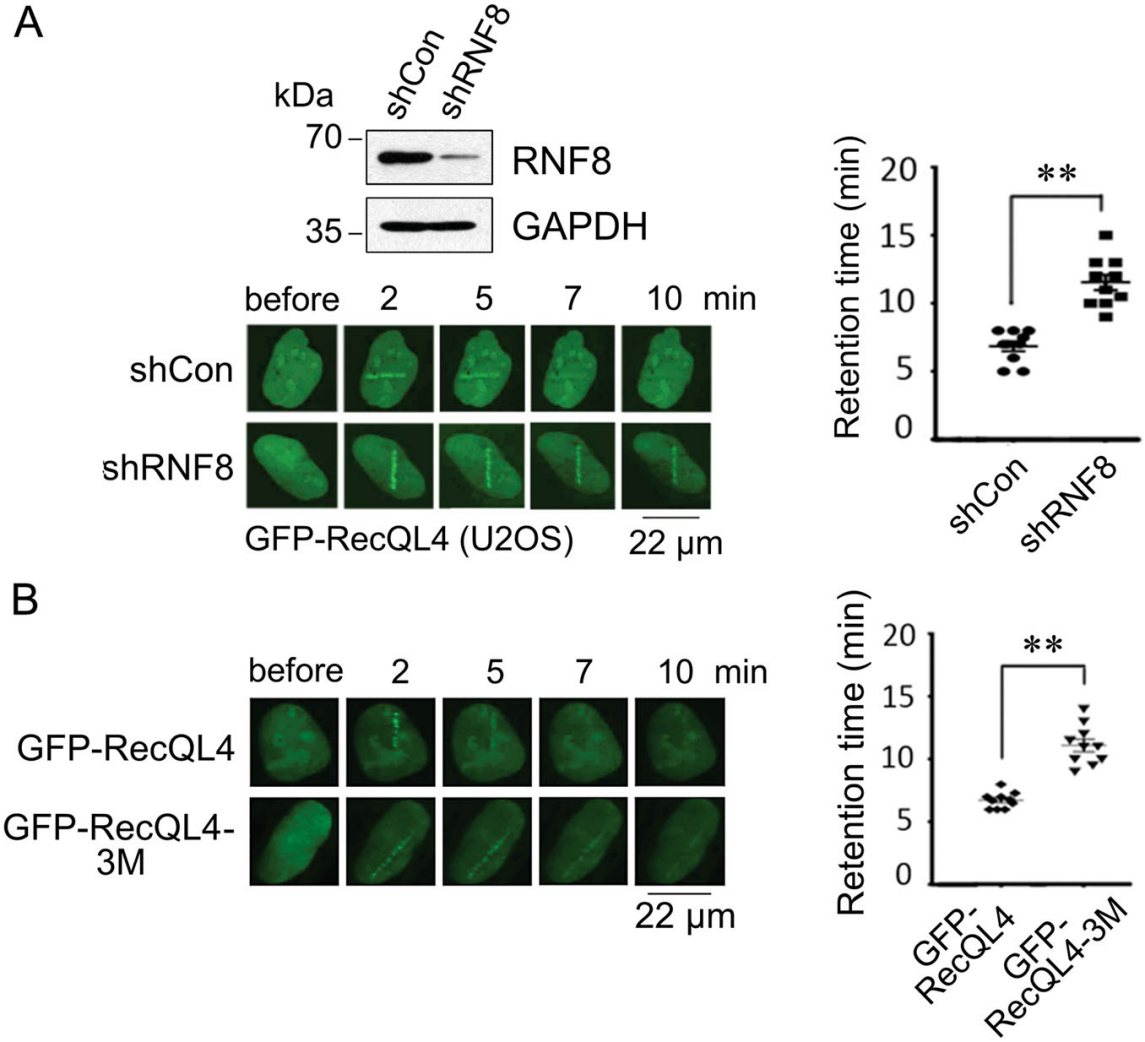
RNF8 promotes RecQL4 dissociation from DSBs. **A** Knockdown of RNF8 significantly inhibits the dissociation of RecQL4 from DSBs. U2OS cells with or without RNF8 depletion were treated with 365-nm micro-point laser. The recruitment of GFP-RecQL4 at DSBs was recorded and the fluorescence density was quantified. **B** The prolonged and enhanced recruitment of RecQL4-3M at DSBs induced by UV micro-point laser. U2OS cells were transfected with GFP-tagged WT RecQL4 or its 3M mutant, and subjected to UV micro-point laser treatment. The recruitment of GFP-RecQL4 or its mutant at DSBs were recorded and the fluorescence density at DSBs was quantified. At least 15 cells were analyzed for each treatment. The data represent mean ± SEM from three independent experiments. **p < 0.01.

Earlier studies showed that RNF8-mediated ubiquitination during DSB repair not only forms a platform for the recruitment of 53BP1 and BRCA1 proteins through modifying histones but also facilitates the removal of repair proteins, such as Ku80, at the DNA damage sites^9^. Therefore, we tested whether RecQL4 ubiquitination level affects its dissociation from DSB sites by using RNF8-deficient and proficient U2OS cells. As shown in Fig. 3A, upon RNF8 knockdown, RecQL4 dissociation from DSB sites was significantly delayed. Consistently, full length of RecQL4 protein with mutated ubiquitination sites (3M, K876R, K1048R, and K1101R) also showed a significantly longer retention at DSB sites compared to WT RecQL4 (Fig. 3B). These findings collectively suggest that RecQL4 ubiquitination mediated by RNF8 promotes its efficient dissociation from the DSB sites.

### RecQL4 ubiquitination status affects both its DSB repair activity and the recruitment of its direct downstream proteins to DSB sites

**Fig. 4:**
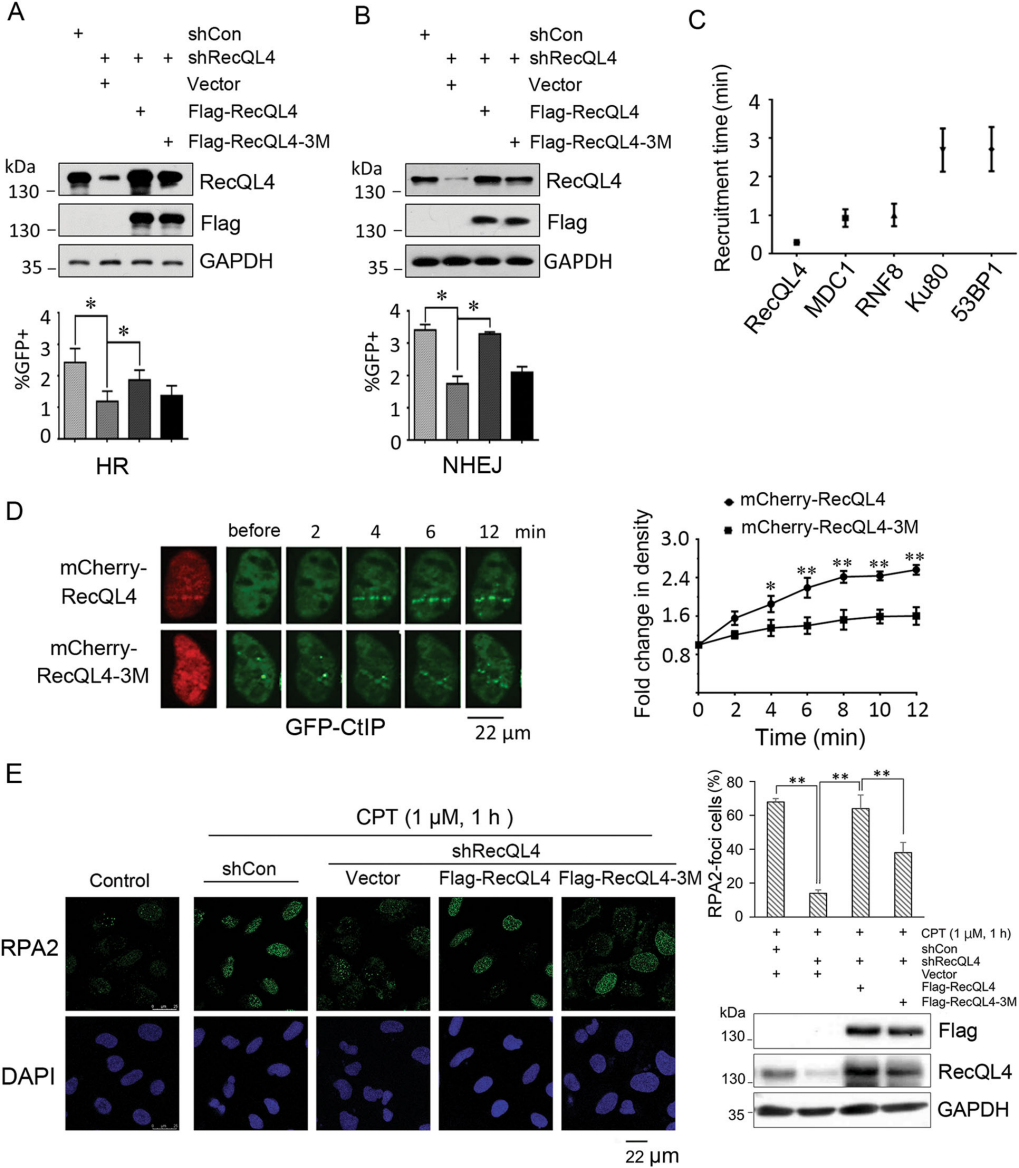
Defective RecQL4 ubiquitination affects its activity in DSB repair and furthers the binding of its direct downstream proteins to DSB sites. **A**, **B** RecQL4 depletion significantly decreased the HR- and NHEJ-mediated DSB repair in U2OS cells quantified by DR-GFP and EJ5-GFP reporter system, respectively. The defective DSB repair was significantly restored by re-introduction of wild-type RecQL4 but not its mutant. The percentage of GFP-positive cells was quantified by Flow cytometry. The data represent mean ± SEM from three independent experiments. (*p < 0.05, Student’s *t* test). **C** Analysis of the time-dependent recruitment of various DSB repair proteins in U2OS cells after micro-point laser treatment. Cells were transfected with GFP-tagged RecQL4, RNF8, Ku80, 53BP1, or mCherry-tagged MDC1 plasmids, followed by the treatment of micro-point laser. The images were captured using time-lapse microscopy. At least 15 cells for each transfection were recorded and analyzed for the earliest time point of protein aggregate formation at DSB track. **D** RecQL4 ubiquitination status affects the recruitment of its directly associated downstream protein-CtIP. RecQL4 was first silenced in U2OS cells followed by transfection with GFP-tagged CtIP and mCherry-tagged wild type RecQL4 or its mutant (3M). The mCherry-positive cells were treated with micro-point laser and the images were captured using microscopy. Both recruitment time and fluorescence density were recorded and at least 15 cells were analyzed. The data represent mean ± SEM from three independent experiments. **p < 0.01. **E** RecQL4 3M mutant interferes with its capacity in processing ssDNA formation at DSB ends estimated by an end resection assay. RecQL4 was first silenced by shRNA infection in U2OS cells which were then transfected with either a control, RecQL4 WT, or 3M mutant for 24 h, followed by the treatment with 1 μM CPT (C9911, Sigma) for 1 h. Cells were fixed for RPA2 (ab2175, Abcam) immunostaining. A total of 200 cells were analyzed and RPA2-foci (>15) positive cells were scored for each individual experiment. Data represent mean ± SEM of three independent experiments (**p < 0.01, the Single Factor Anova test). Fluorescence images were captured using a LEICA TCS SP8 confocal microscope system. Scale bar, 22 μm.

RecQL4 has the demonstrated role(s) in the HR/NHEJ-mediated DSB repair^20^. Here, we tested the impact of RecQL4 ubiquitination on DSB repair by utilizing HR and NHEJ reporter assays that were described previously^21^. In agreement with previous findings^13,15,20^, it was observed that RecQL4 silencing in DR-GFP and EJ5-GFP U2OS cells led to a significant decrease in HR and NHEJ repair efficiency by 65.0% and 67.2%, respectively, compared to empty vector treated mock cells (Fig. 4A, B). Interestingly, this defective DSB repair activity was mostly rescued by introducing wild type RecQL4, instead of its ubiquitination mutant, indicating that RecQL4 activity in DSB repair depends on its status of ubiquitination modification.

Since the sites of DSB repair are expected to contain numerous factors, sequential assembly and removal of repair factors are critical for the completion of DSB repair in a timely fashion. To determine the sequential order of assembly of RecQL4 relative to other DSB repair-associated proteins (MDC1, RNF8, 53BP1, and Ku80), the time-dependent recruitment of these factors was examined at the DSB sites induced by micro-point laser treatment. RecQL4 was observed to be the earliest one with an average of 13 s, while other factors were recruited much later than RecQL4 ranging from 1 to 2.5 min (Fig. 4C).

We then determined whether RecQL4 depletion affects the recruitment of its downstream proteins at DSBs. Ku80 showed a marked decrease of recruitment at DSB sites upon RecQL4 silencing (Supplementary Fig. 4), which is consistent with their direct interaction reported previously^13^, while the other three proteins (MDC1, RNF8, and 53BP1) whose fluorescence density and recruitment times at DSB sites were not affected by RecQL4 depletion (Supplementary Figs. 5–7). Specifically, recruitment of RNF8 to DSB sites is not mediated by RecQL4, even though both proteins co-localize at the DSB sites.

Effect of RecQL4 ubiquitination status on the recruitment of its direct downstream factors, such as CtIP^12^ was next examined. For this purpose, U2OS cells were first depleted of RecQL4 expression using Adeno virus-mediated RecQL4 shRNA^22^. The RecQL4 silenced cells were subsequently reconstituted with either mCherry-WT-RecQL4 or mCherry-RecQL4-3M, together with GFP-CtIP. The DSBs were induced in the mCherry-positive cells by micro-point laser. The data showed that the time-dependent recruitment of CtIP at DSBs was not drastically altered, but their fluorescence density at DSBs was significantly decreased in RecQL4-3M cells compared to WT control (Fig. 4D). Similarly, RecQL4-3M reconstituted cells had a significant decrease in both Ku80 density at DSBs induced by micro-point laser and proportion of Ku80 foci (>15) positive cells by X-ray irradiation compared to the cells with re-expression of WT RecQL4 (Supplementary Figs. 8, 9).

We then employed a DNA end resection assay using RPA2 foci formation as a surrogate marker^23^ to determine whether prolonged retention of RecQL4 3M at DSBs hinders CtIP recruitment through affecting single-strand DNA (ssDNA) processing. U2OS cells with RecQL4 depletion by adenoviral vector-mediated shRNA were reconstituted with either WT RecQL4 or 3M mutant, followed by the treatment of 1 µM Camptothecin (CPT) for 1 h. The result showed that upon CPT challenging, RecQL4 depletion significantly decreased the percentage of RPA2-positive (>15 foci) cells in relative to shControl, which could be mostly rescued by reconstitution of WT RecQL4, but not 3M mutant (Fig. 4E). This finding suggests that the delayed dissociation of RecQL4 3M from DSBs interferes with its capability in processing ssDNA formation, and further the recruitment of its direct downstream DSB repair proteins, such as CtIP and Ku80.

### WRAP53β-RNF8-RecQL4 forms a triple complex coordinately regulating DSB repair

**Fig. 5:**
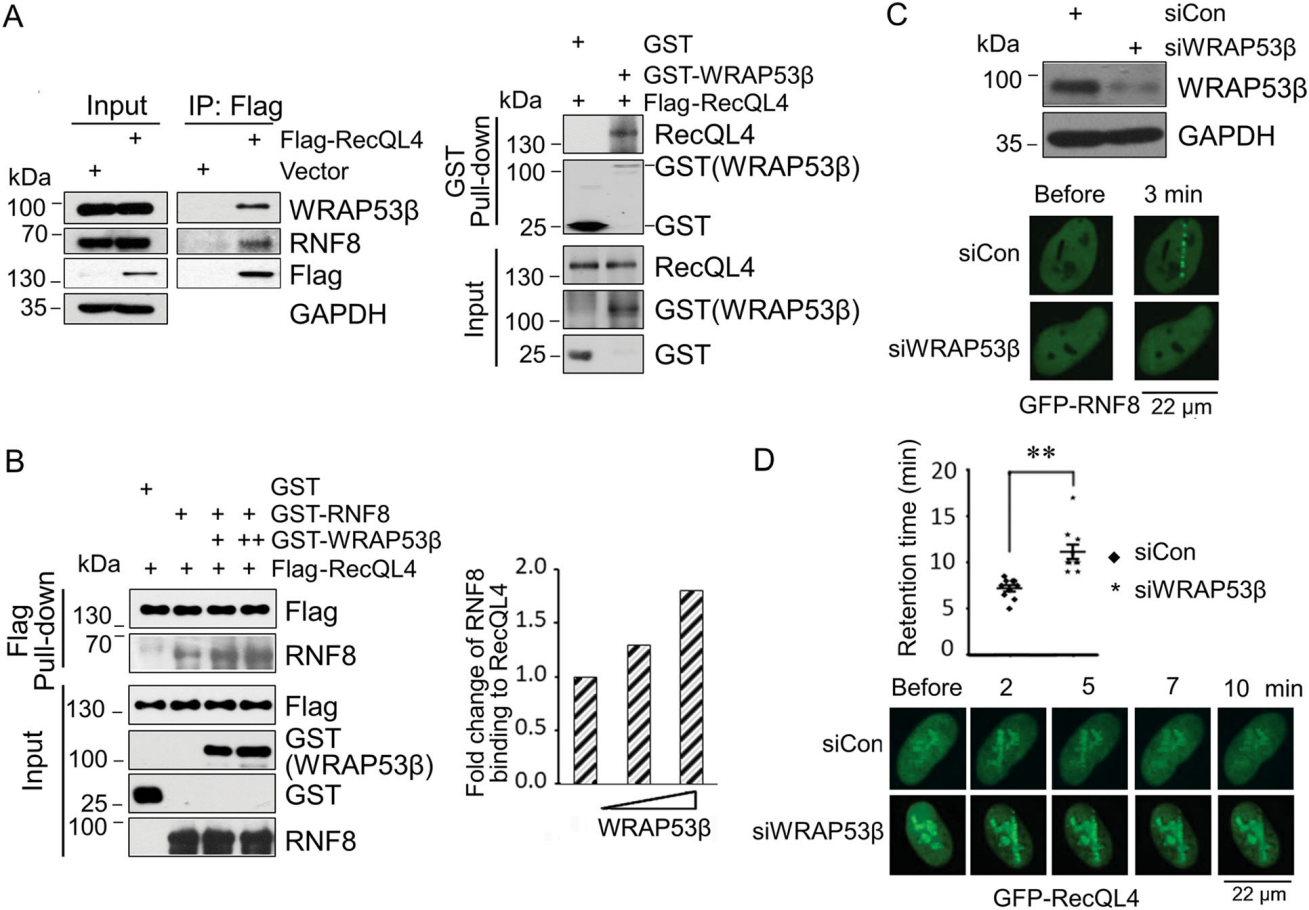
WRAP53β promotes the association between RNF8 and RecQL4. **A** RecQL4 interacts with WRAP53β. Endogenous WRAP53β was present in Flag immunoprecipitated fractions using the lysate of HEK293 cells transfected with Flag-RecQL4. Direct interaction between RecQL4 and WRAP53β was further demonstrated by an in vitro pull-down assay using the purified recombinant GST-WRAP53β to pull down Flag-RecQL4. **B** A markedly enhanced interaction between RecQL4 and RNF8 was observed upon the increased input of WRAP53β protein assessed by an in vitro Flag pull-down assay, followed by the western blotting analysis. **C** Dynamic recruitment of GFP-RNF8 to DSBs induced by UV micro-point laser in WRAP53β silenced U2OS cells relative to scrambled siRNA-transfected cells. **D** Dynamic recruitment of GFP-RecQL4 to DSBs in scrambled siRNA or WRAP53β siRNA-transfected U2OS cells.

Previous reports demonstrated that scaffold protein WRAP53β targets RNF8 to DSBs by facilitating protein–protein interactions^6^. As reciprocal silencing of RNF8 and RecQL4 did not affect their recruitment kinetics except for a prolonged retention of RecQL4 at DSBs in RNF8-depleted cells (Fig. 3A), we, therefore, wished to test the possibility that WRAP53β through its interaction with RecQL4 facilitates the association of RecQL4 with RNF8 at DSBs. The co-IP and in vitro GST pull-down experiments proved that RecQL4 interacts with endogenous WRAP53β and both proteins exhibit a direct association (Fig. 5A). In vitro Flag pull-down assay further demonstrated that with increasing the input of GST-WRAP53β protein, the interaction of RecQL4 with RNF8 was substantially enhanced (Fig. 5B), suggesting that WRAP53β may serve as a docking platform facilitating the interaction between RecQL4 and RNF8, and these three proteins form a trio exerting an important role during DSB repair.

We next determined whether WRAP53β knockdown affects the RNF8 recruitment and further RecQL4 dissociation from DSBs. WRAP53β was silenced in U2OS cells by transfection with WRAP53β siRNA using the siRNA sequences reported previously^6^ and the efficiency of WRAP53β silencing was assessed by western blotting. As expected, the recruitment of GFP-RNF8 to DSBs was nearly absent in WRAP53β-depleted cells, relative to control siRNA-treated cells that showed a strong signal at 3 min after micro-point laser treatment (Fig. 5C). While GFP-RecQL4 at DSBs was completely dissociated from DSBs after 7 min post micro-point laser treatment in control siRNA cells, persistence of GFP-RecQL4 was still observed after 10 min of at the DSB sites in WRAP53β silenced cells (Fig. 5D). Although WRAP53β silencing did not affect the recruitment of RecQL4 to the DSB sites (Fig. 5D), WRAP53β silencing induced a defective recruitment of RNF8 to DSBs, leading to a defective dissociation of RecQL4 from chromatin at DSBs. Therefore, the coordinated role of WRAP53β-RNF8-RecQL4 signaling axis is critical for the efficiency of RecQL4 in mediating the DSB repair process.

## Discussion

Ubiquitination plays a pervasive role in the dynamic assembly and disassembly of DDR proteins on the DNA damage sites, where the E3 ligase RNF8 plays an essential role in initiating a cascade of ubiquitination in response to DNA double-strand breaks. RNF8/RNF168 has been shown not only to promote the recruitment of many downstream factors, such as 53BP1, RAP80, and BRCA1^7^ but also to mediate extraction of some proteins, such as Ku80^9^, from the chromatin at DSB sites. RecQL4 is a critical member of human RecQ helicase family and is implicated in both HR and NHEJ-mediated DSB repair pathways^12,14^. It was shown that an enhanced activity of RecQL4 in DSB repair is augmented by its ubiquitination mediated by DDB1-CUL4A E3 ligase, the process that can be promoted by CDK1/2-mediated RecQL4 phosphorylation^15^. However, how RecQL4 is released from the DSB sites after it fulfills its task has remained elusive. Here, we demonstrate that RecQL4 ubiquitination by RNF8 facilitates its dissociation from DSB sites. RecQL4 recruitment to DSB sites is a much earlier event relative to other DNA repair proteins including RNF8. Knockdown of RNF8 expression not only induces a significantly prolonged retention of RecQL4 at DSB sites but also interferes with the subsequent recruitment of its downstream proteins, such as CtIP, eventually resulting in a defective DSB repair. In support of the previously reported role of WRAP53β in mediating RNF8’s binding to DSB sites^6^, we also observed that WRAP53β depletion results in a reduced recruitment of RNF8 to the DSB sites. Furthermore, WRAP53β forms a complex with both RecQL4 and RNF8, and markedly enhances their interaction at the DSB sites. Our findings strongly suggest that WRAP53β-RNF8-RecQL4 forms a heterotrimeric protein complex at the DSB sites to regulate the DSB repair activity of RecQL4.

It has been reported that ultraviolet lights UV-C (200–280 nm) and UV-B (280–320 nm) primarily generate UV photo-lesions of cyclobutane pyrimidine dimers (CPDs) and 6–4 photoproducts (6–4PP)^24^, which could indirectly result in secondary DNA damages, such as ssDNA and DSB, through a nucleotide excision repair (NER)-dependent mechanism^25^. However, UVA micro-irradiation at 365 nm has been found to predominantly induce base damage and an aberrant DSB response^26,27^, and has become as a valuable tool to study the spatiotemporal dynamics of DSB damage recognition/response^28–30^. Nevertheless, the DSBs at UVA track might represent a chemically distinct and complex damages, compared to the ones induced by high/low linear energy transfer (LET) irradiation^27^. We have employed low LET X-ray irradiation to induce DSBs, and demonstrated the following findings upon X-ray treatment: a substantially enhanced level of RecQL4 ubiquitination (Supplementary Fig. 10), co-localization of endogenous RecQL4 and RNF8, and significantly lower proportion of Ku80 foci (>15) positive cells under RecQL4-3M reconstitution condition. These data, in corroboration with the findings from UV micro-point laser, suggest that modulation of RecQL4 ubiquitination by RNF8 is DSB-dependent.

RNF8 is the first E3 ubiquitin ligase that is rapidly accumulated at the DSB sites where the interaction with ATM-phosphorylated MDC1 is required for its recruitment^7,31^. In addition, the scaffold protein WRAP53β has been demonstrated to rapidly localize at the DSB sites in an ATM- and MDC1-dependent manner. It then targets RNF8 to DNA lesions through enhancing the interaction between RNF8 and its upstream partner MDC1^6^. WD40 domain-containing WRAP53β serves as a scaffold protein to facilitate protein–protein interactions functioning in splicing, telomere elongation, and DSB repair^6^. Genetic mutation of WRAP53β results in several human disorders, including dyskeratosis congenita, premature aging, and cancer predisposition^32^. Similar to WRAP53β, RecQL4 plays diverse regulatory roles in DNA metabolism (replication, transcription, recombination, and damage repair), telomere maintenance, and cell cycle progression^33,34^. Here, a direct physical interaction of WRAP53β with RecQL4 has been demonstrated. Based on or experimental evidence, we speculate that WRAP53β may serve as an important platform for potentiating the interaction of RecQL4 with other critical factors, such as RNF8, through which to regulate the DSB repair activity of RecQL4.

RNF8 can initiate both K48- and K63-linked ubiquitin chain formation on its substrates at the DSB sites. The former one mediates the removal and degradation of Ku80 of NHEJ pathway from DSB sites, while the later one can be recognized by RNF168 that can polyubiquitinate a list of downstream DSB repair-associated proteins, such as 53BP1 and BRCA1 of HR pathway^9^. Additionally, both K6- and K63-based ubiquitination have been detected to be present at the DNA damage sites^35^, and RNF8 has been demonstrated to mainly mediate K6-linked ubiquitin conjugate on NBS1 contributing to an efficient and stable binding of Nbs1 to DSBs^17^. RNF8 could also promote K11-linkage conjugates on damaged chromatin, including histone H2A/H2AX to regulate DNA damage-induced transcriptional silencing, which is distinct from Lys63-linkage ubiquitin mainly responsible for the recruitment of DNA damage repair proteins^36^. We found that RNF8 can target RecQL4 preferentially through mediating K6-, K27-, and K29-linkage ubiquitination of RecQL4, which are the noncanonical forms of ubiquitination and have been shown to participate in the regulation of either protein stability^37,38^ or proper activation of DNA damage response^17,39^. Meanwhile, silenced or overexpressed RNF8 expression did not affect the stability of RecQL4 protein (Supplementary Fig. 11). Thus, RNF8-mediated RecQL4 ubiquitination mainly regulates its dissociation from DSBs.

The complex of ubiquitin-dependent unfoldase/segregase VCP/p97 and deubiquitinase Ataxin 3 (ATX3) has been shown to facilitate RNF8 chromatin extraction^18,40^. Consistently, we also observed that RNF8 exhibited a significantly earlier recruitment versus much stronger recruitment density at DSBs upon VCP/p97 knockdown (Supplementary Fig. 12A). In contrast, RecQL4 retention at DSBs was significantly shortened after VCP/p97 silencing (Supplementary Fig. 12B), suggesting that the enhanced RNF8 retention at DSBs after VCP/p97 silencing facilitates the removal of RecQL4 from DSBs. This data supports the role of RNF8 in regulating the dissociation of RecQL4 from DSBs through a ubiquitination-dependent mechanism.

RecQL4 has been shown to promote CtIP recruitment at DSBs where both are involved in the single-strand DNA (ssDNA) processing^12^, which is supported by our finding that RecQL4 depletion significantly decreased the recruitment of CtIP at DSBs induced by UV micro-point laser. It is well-documented that accuracy in sequential assembly or disassembly of repair factors or proteins at DSB sites is prerequisite for an efficient DSB repair activity^41^, and RecQL4 was found to be recruited to the DSB sites at a very early time point. Therefore, timely dissociation of RecQL4 from DSB sites is expected to provide free accessible DNA ends facilitating the binding of its downstream factors. Thus, the considerably delayed/attenuated dissociation of RecQL4-3M from DSBs is likely to hinder rather than promote the recruitment of its direct downstream factors, such as CtIP. In support, observations from the rescue assays illustrated that in relative to WT RecQL4, reconstitution of RecQL4-3M led to a significantly lower recruitment of CtIP at DSB track, which is further substantiated by a DNA end resection assay showing that RecQL4 3M significantly interferes with the ssDNA formation as judged by the less proportion of RPA2 foci positive cells relative to WT RecQL4. Similar to the observed effect of RecQL4-3M on CtIP, we also observed that RecQL4-3M reconstitution resulted in a substantially decreased recruitment density of Ku80 at DSBs and much less proportion of Ku80 foci positive cells after X-ray irradiation. Although it is suggested that the binding of Ku70/Ku80 complex to DSBs was the first step in NHEJ-associated repair activity^42^, both our findings and other report demonstrated that RecQL4 might bind damaged DNA much earlier than Ku complex^13^. Therefore, the persistence of RecQL4 3M at DSB sites owing to defective ubiquitination interferes with the binding of its direct downstream factors, including CtIP and Ku80.

RNF8 appears to be a versatile E3 ligase potently initiating different lysine site-linkage ubiquitination of multiple key players, like RecQL4 in our study, in NHEJ- and HR-mediated DSB repair pathways. Moreover, our study has identified a novel heterotrimeric complex of RecQL4, RNF8, and WRAP53β that regulates the efficiency of DSB repair. Future studies will determine whether or not this complex also functions in other DNA repair pathways.

## Materials and methods

### Cell culture and reagents

Human U2OS osteosarcoma cells and HEK293 human embryonic kidney cells were purchased from ATCC, and grown in DMEM containing 10% Fetal Bovine Serum (BioWest) at 37 °C in humidified 5% CO_2_ incubator. These cells were tested for negative mycoplasma and authenticated using Short Tandem Repeat (STR) profiling analysis by Shanghai Biowing Applied Biotechnology Co. LTD, Shanghai, China. Primary antibodies used in this study were listed as the following: Flag (F1804, Sigma), RecQL4 (25470002, Novus Biologicals), RNF8 (14112-1-AP, Proteintech); GAPDH (MAB374, Millipore); 53BP1 (A300–272A, Bethyl), CtIP (A300-488A, Bethyl), Ku80 (#2753, Cell Signaling Technology), Rad51 (sc-8349, Santa Cruz Biotechnology), GST (10000-0-AP, Proteintech), WRAP53β (14761-1-AP, Proteintech).

### Plasmids and RNA interferences

pFlag-CMV4-RecQL4, Flag-RecQL4-NT, Flag-RecQL4-HD, Flag-RecQL4-CT, and pcDNA3.0-HA-RNF8 were described previously^22,43^. RNF8 and RecQL4 fragments were subcloned into pEGFP-c1B. mCherry-tagged RNF8 was subcloned into pFlag-CMV4. The coding DNA sequences of WRAP53β were amplified by PCR with cDNA library of human U2OS cells and subcloned into p3×Flag-CMV-10 (Sigma). The coding DNA sequences of RNF8 and WRAP53β were amplified by PCR and subcloned into pGEX-6P-1 (GE Healthcare). RecQL4 mutants were generated with QuickMutation™ Site-Directed Mutagenesis Kit (Beyotime) according to the manufacturer’s protocol. To generate shRNF8 adenovirus constructs, a 19-mer RNA shRNA (ACATGAAGCCGTTATGAAT, accession no. NM_003958.3) was recombined into pAdEasy-1 vectors and RNF8-knockdown recombinant adenovirus was generated in HEK293 cells^22^.

### Generation of DSBs

Micro-point laser treatment and conditions for time-lapse microscopy were performed with an UltraVIEW VOX (PerkinElmer). Living Cells cultured at 37 °C, 5% CO_2_ were irradiated with two pulses of the 365-nm laser beam at 80% power, and then the dynamic recruitments processes were recorded using Nikon TIE microscope and UltraVIEW VOX imaging system.

### Co-immunoprecipitation assay

Denaturing immunoprecipitation was performed following the procedures described previously^7,22^. Briefly, cells containing expression proteins were lysed in buffer (20 mM Tris-HCl, pH7.5, 150 mM NaCl, 0.5% NP-40, 0.5% SDS, 1 mM EDTA, 1 mM PSMF) and dissolved by sonication. After heating at 95 °C, the extracts were centrifuged for 15 min at 12,000 g. Then, the flag-tagged proteins were immunoprecipitated with anti-Flag M2 agarose beads and analyzed by western blotting. For non-denaturing immunoprecipitation, cells were lysed in an SDS-free buffer. For western blotting, cells were lysed in RIPA (Radio Immuno Precipitation Assay) containing protease inhibitors.

### Protein expression and purification

Flag-RecQL4, GST-RecQL4 CT-Flag, GST-RecQL4 CT-Flag 3M mutant, and GST-RNF8 were purified as described previously^22^. Briefly, various RecQL4 protein expression was induced in *E. coli* Rosetta (DE3) cells at 16 °C in the presence of 0.1 mM IPTG overnight, and purified as previously described^22^ after collecting and lysing the cells in a buffer (50 mM potassium phosphate, pH8.0, 300 mM NaCl, 10% glycerol, protease inhibitors). GST-RNF8 and GST-WRAP53β expressing cells were lysed in buffer (10 mM Tris-HCl pH 7.4, 200 mM NaCl) containing protease inhibitors, and applied to GST beads (GE healthcare) for binding recombinant proteins.

### In vitro pull-down assay

For GST pull-down assay, GST-RNF8 purified from BL21 *E. coli* bacteria was first immobilized on glutathione Sepharose 4B at 4 °C overnight, followed by incubation with Flag-RecQL4 at 4 °C for 4 h. For Flag pull-down assay, Flag-RecQL4 was immobilized with M2 beads at 4 °C overnight, washed, and then GST fusion proteins were added and incubated with Flag-RecQL4 protein immobilized Flag M2 beads at 4 °C for another 4 h. Sepharose beads were then washed and boiled in 2 × SDS loading buffer. Samples were subjected to western blotting with indicated antibodies.

### Ubiquitination assay in vitro

RecQL4 ubiquitination assays were performed as previously described^43^. Briefly, purified GST-RecQL4-CT-Flag or GST-RecQL4-CT (3M)-Flag was immobilized on Flag-M2 beads, and then incubated with E1, E2 (UbcH5c), E3 (GST-RNF8), and 1 μg HA-Ub at 37 °C in 30 μL reaction buffer (50 mM Tris-HCl, pH 7.5, 5 mM MgCl_2_, 2 mM ATP, 1 mM DTT) for 3 h. After washing three times with reaction buffer, the bound protein was eluted and subjected to western blotting analysis with anti-HA antibody.

### HR and NHEJ assay

DR-GFP U2OS cells and EJ5-GFP U2OS cells^44^ were treated with shRNA or control shRNA for 48 h, then transfected with pCBA-I-SceI expression plasmid or control plasmid pCBA. Cells were harvested 72 h later and analyzed by flow cytometry. DR-GFP U2OS cells or EJ5-GFP U2OS cells were treated with shRNA for 36 h to silence endogenous RecQL4, and then transfected with 1.5 μg Flag-RecQL4, Flag-RecQL4 3M (K to R mutant) or vector, and the plasmids expressing I-SceI endonuclease. Cells were harvested at 72 h and analyzed by flow cytometry. HR or NHEJ repair efficiency was expressed as a percentage of GFP-positive cells.

### Statistics

For HR and NHEJ assays, data were presented as the mean ± SEM of three independent experiments. For the real-time recruitment of DDR proteins at DSBs, data are presented as the mean ± SEM of three independent experiments each with the analysis of a minimum of 15 cells (*n* = 15). The measurements of fluorescence intensity of proteins at laser tracks (DSB-containing nuclear regions) were performed essentially as described previously^41^. Statistical analyses were performed with the Student’s *t* test. The *p* value < 0.05 was considered as statistically significant.

## Supplementary information

Supplementary Figure 1

Supplementary Figure 2

Supplementary Figure 3

Supplementary Figure 4

Supplementary Figure 5

Supplementary Figure 6

Supplementary Figure 7

Supplementary Figure 8

Supplementary Figure 9

Supplementary Figure 10

Supplementary Figure 11

Supplementary Figure 12
