# Supplementary figures and images for: RNF8 ubiquitinates RecQL4 and promotes its dissociation from DNA double strand breaks
Source: Oncogenesis. 2021 Mar 5;10(3):24. doi: 10.1038/s41389-021-00315-0 (PMC7935965; doi:10.1038/s41389-021-00315-0)

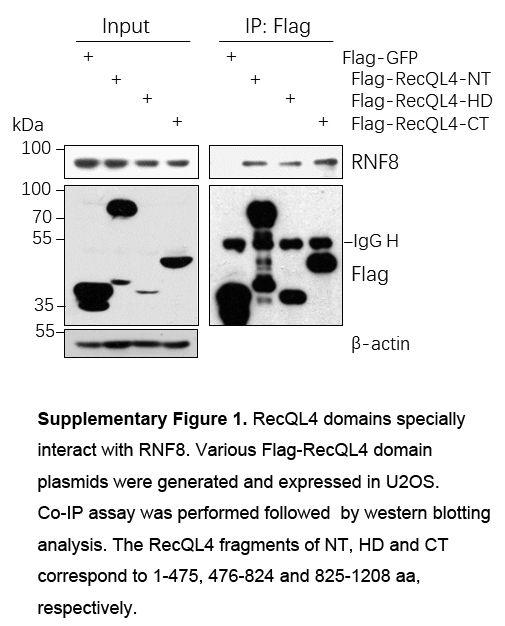

Supplement: Supplementary file 1 — Supplementary Figure 1 [file 41389_2021_315_MOESM1_ESM.tif]

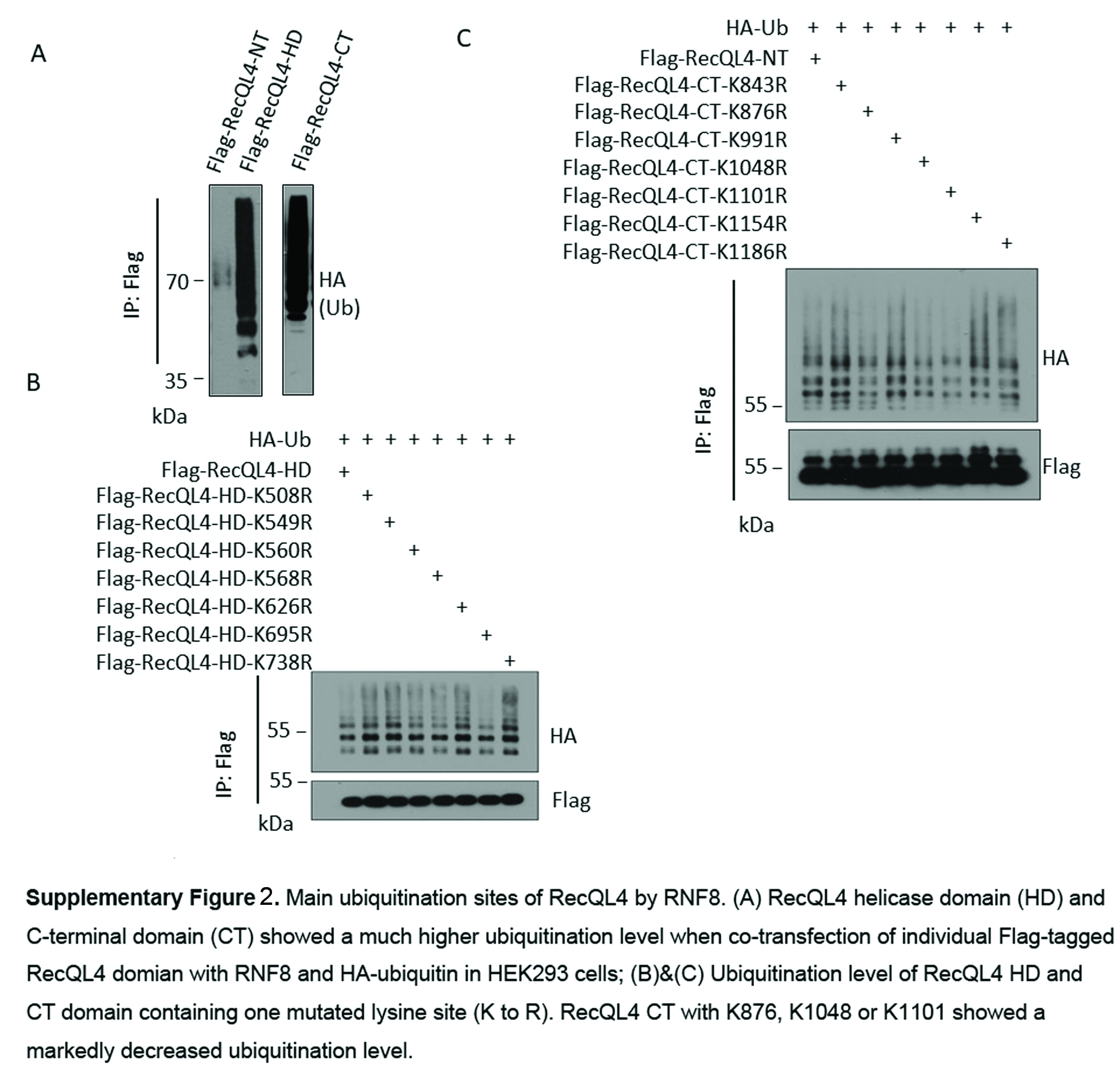

Supplement: Supplementary file 2 — Supplementary Figure 2 [file 41389_2021_315_MOESM2_ESM.tif]

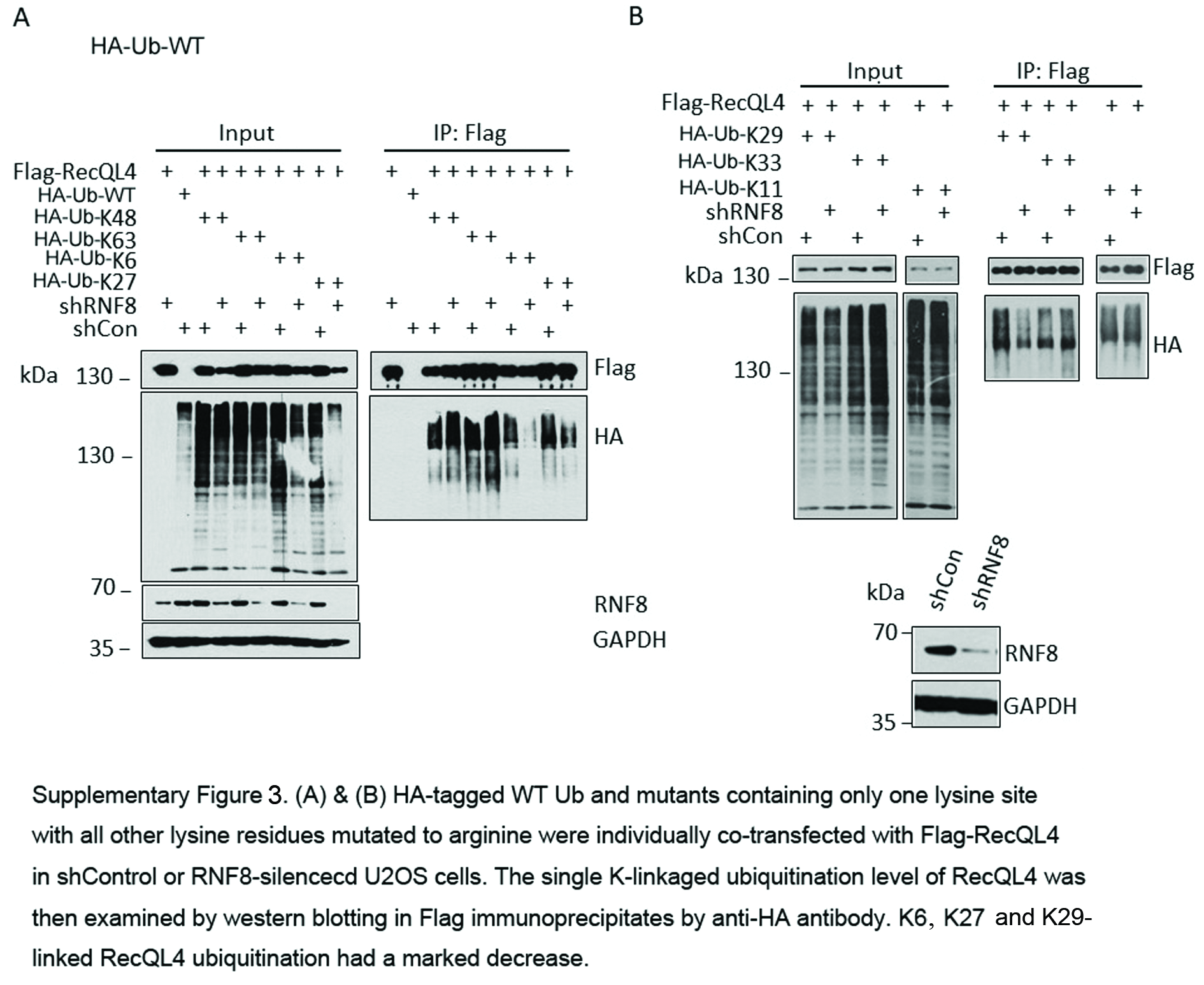

Supplement: Supplementary file 3 — Supplementary Figure 3 [file 41389_2021_315_MOESM3_ESM.tif]

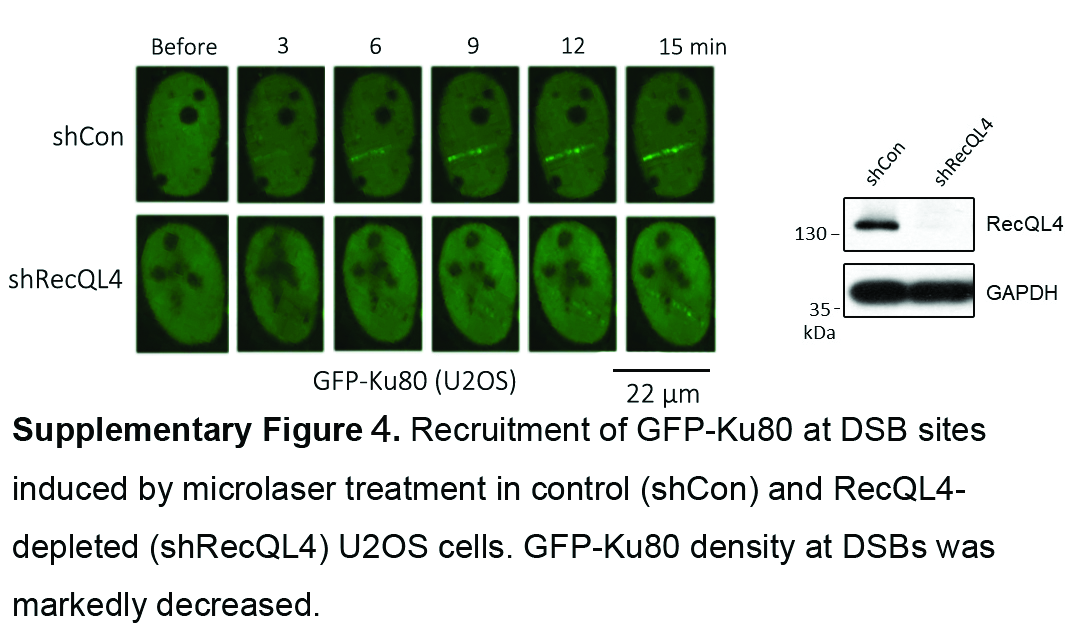

Supplement: Supplementary file 4 — Supplementary Figure 4 [file 41389_2021_315_MOESM4_ESM.tif]

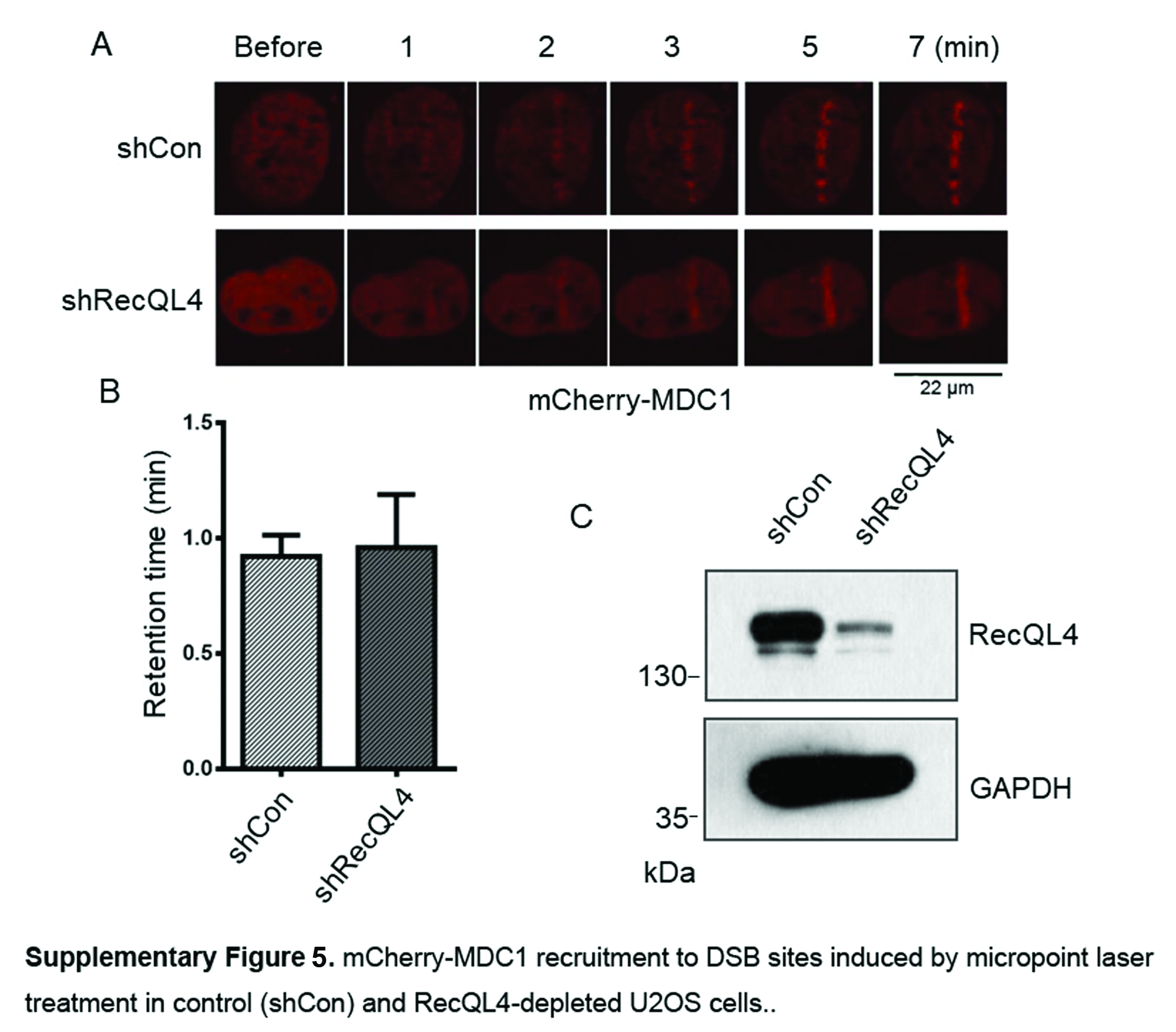

Supplement: Supplementary file 5 — Supplementary Figure 5 [file 41389_2021_315_MOESM5_ESM.tif]

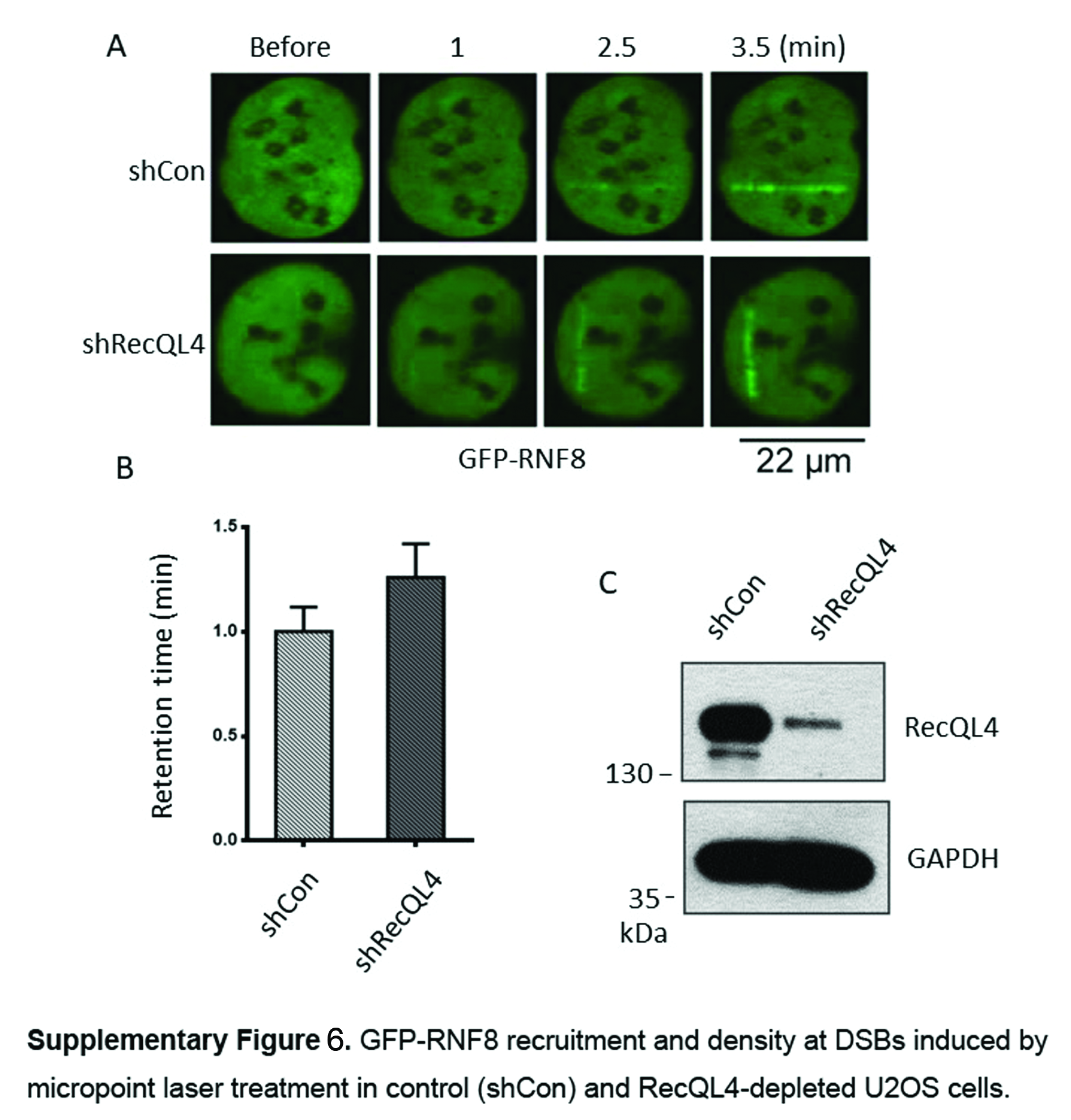

Supplement: Supplementary file 6 — Supplementary Figure 6 [file 41389_2021_315_MOESM6_ESM.tif]

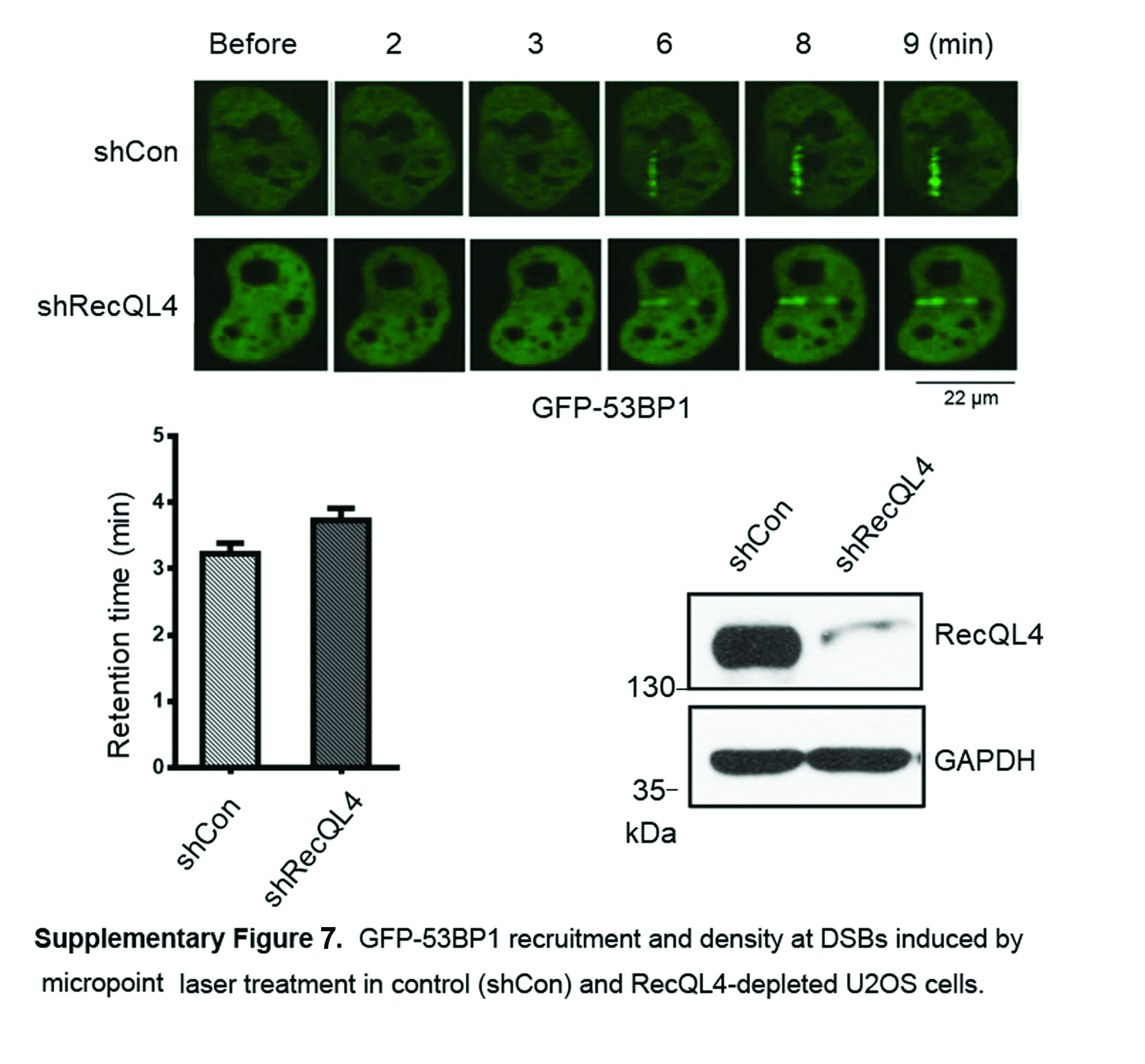

Supplement: Supplementary file 7 — Supplementary Figure 7 [file 41389_2021_315_MOESM7_ESM.tif]

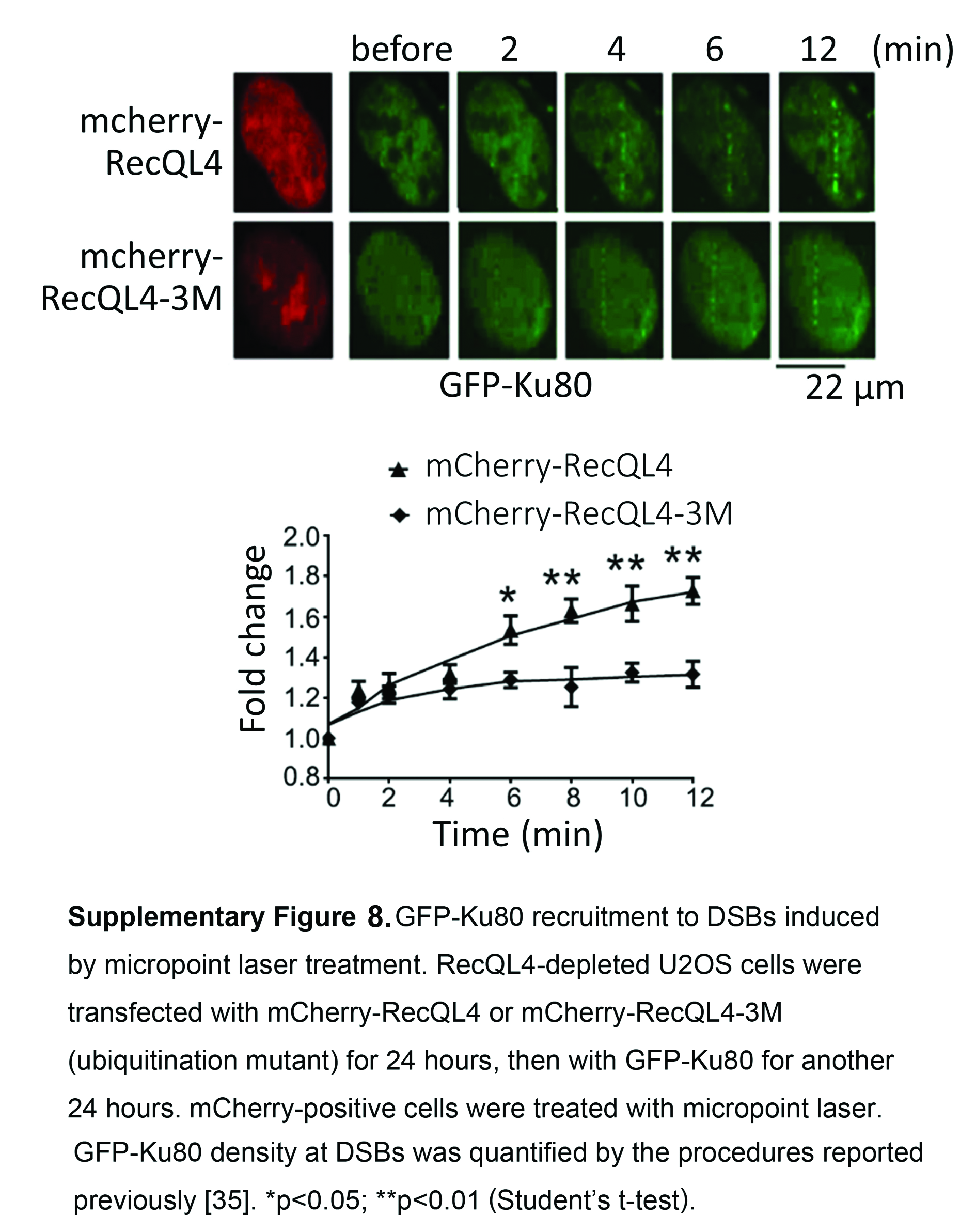

Supplement: Supplementary file 8 — Supplementary Figure 8 [file 41389_2021_315_MOESM8_ESM.tif]

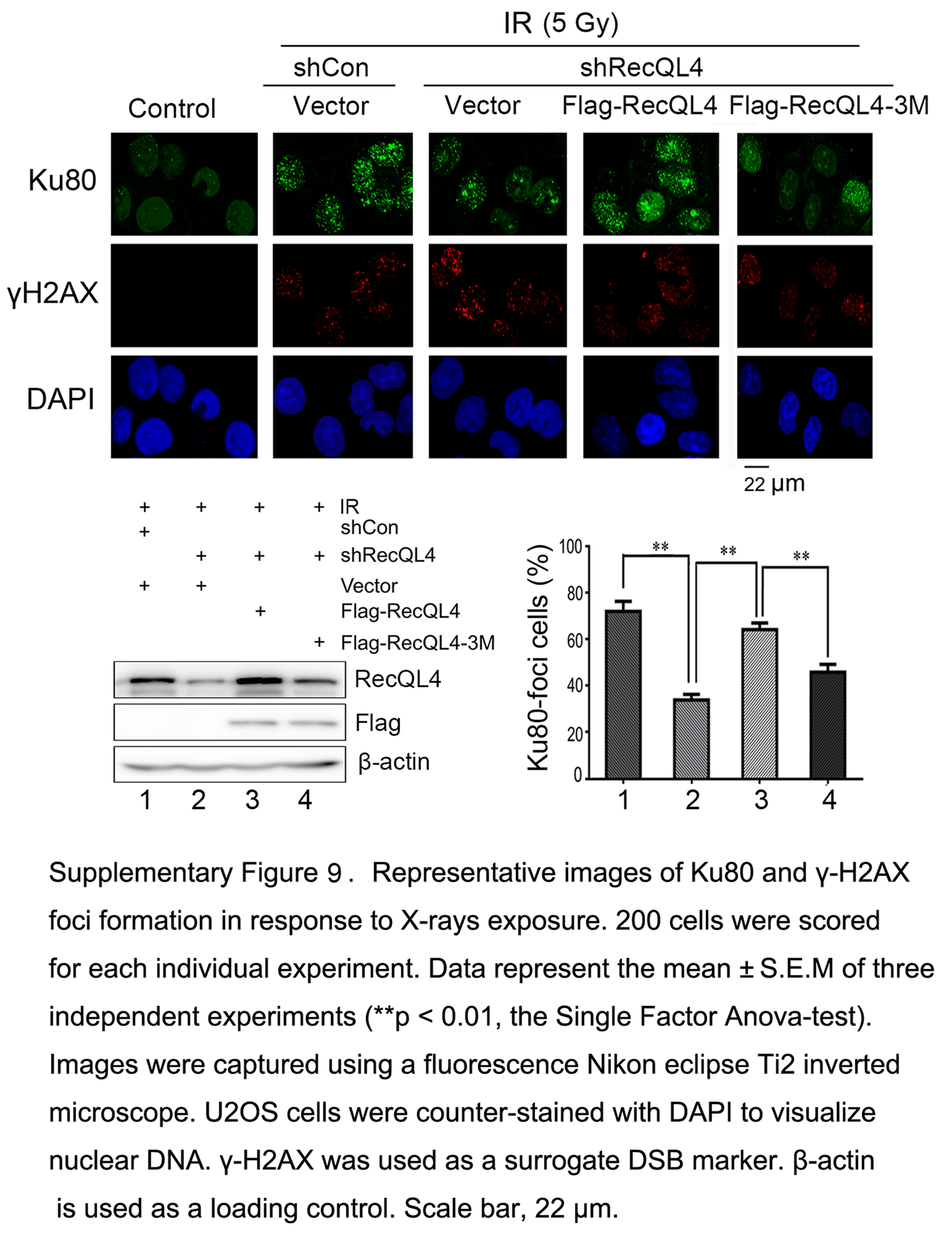

Supplement: Supplementary file 9 — Supplementary Figure 9 [file 41389_2021_315_MOESM9_ESM.tif]

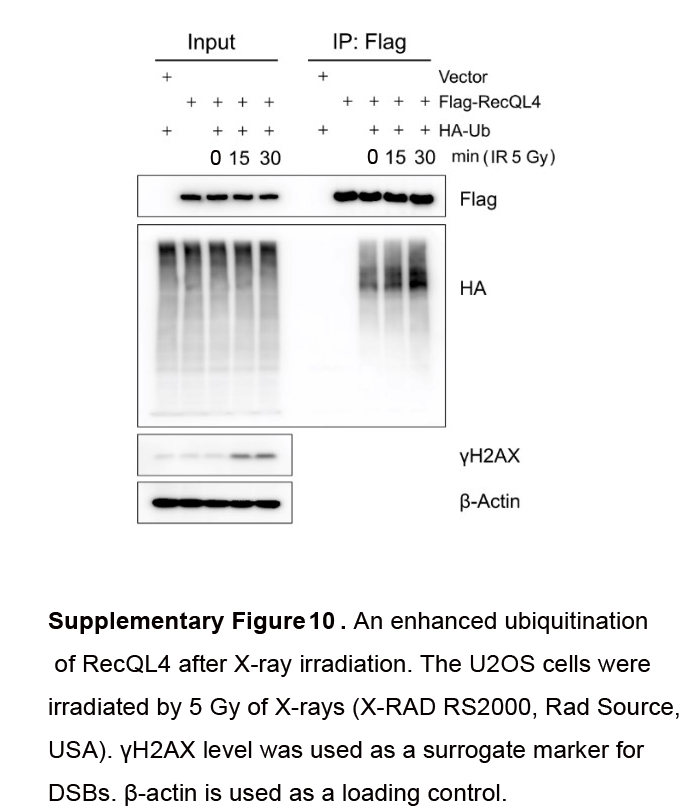

Supplement: Supplementary file 10 — Supplementary Figure 10 [file 41389_2021_315_MOESM10_ESM.tif]

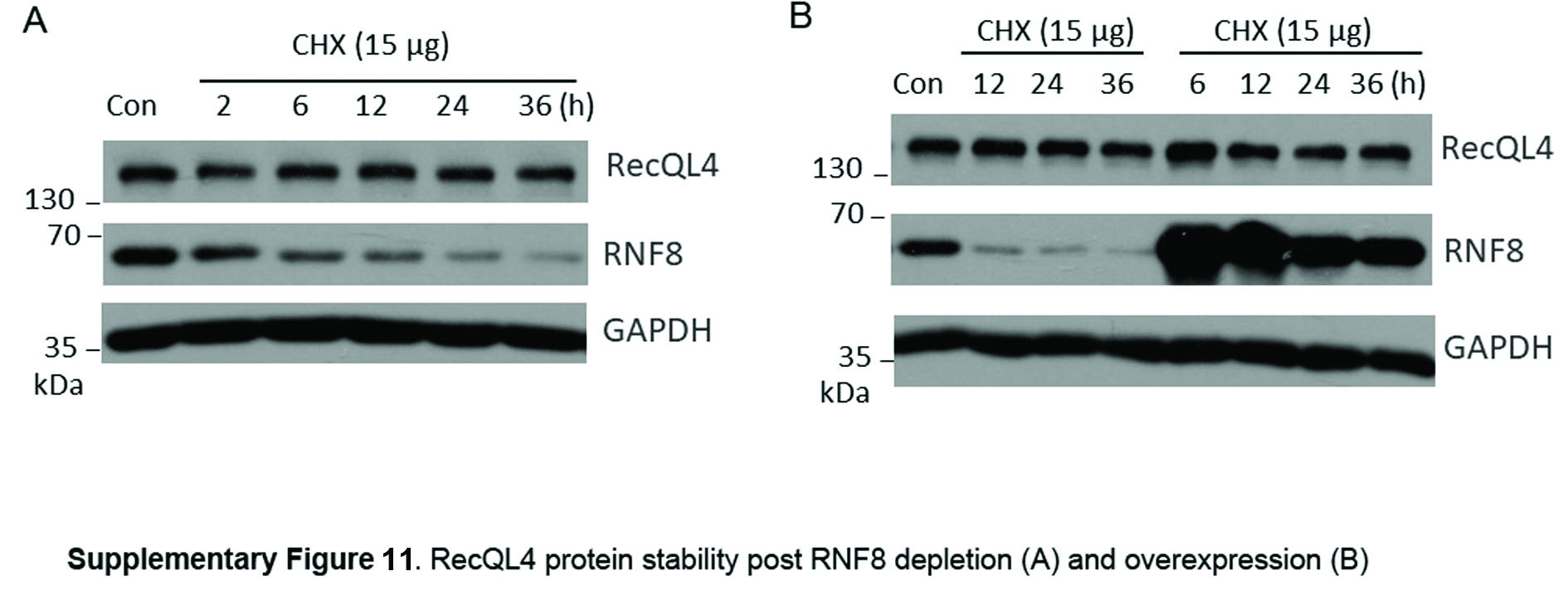

Supplement: Supplementary file 11 — Supplementary Figure 11 [file 41389_2021_315_MOESM11_ESM.tif]

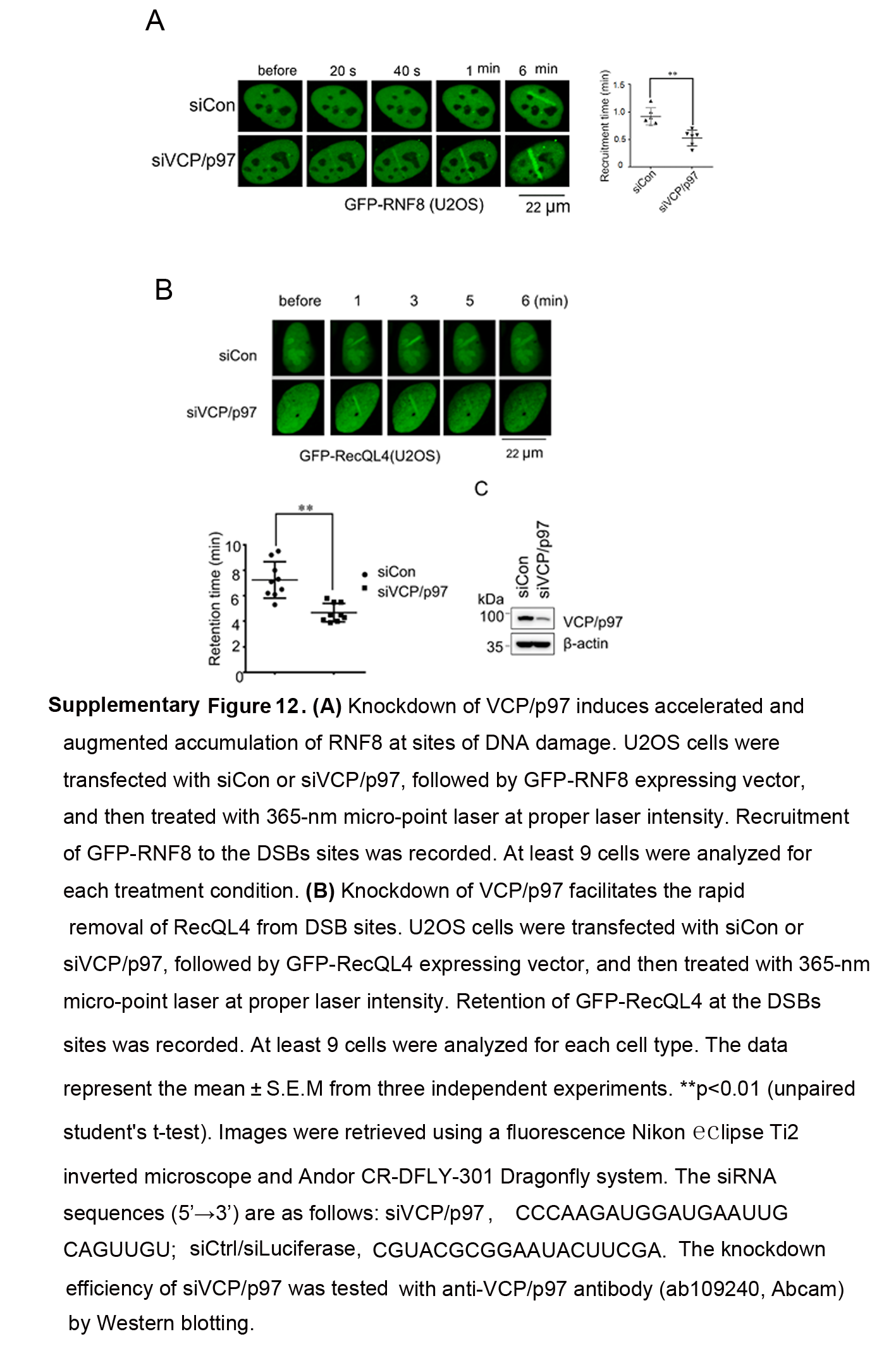

Supplement: Supplementary file 12 — Supplementary Figure 12 [file 41389_2021_315_MOESM12_ESM.tif]
